# The design of an observational study of hypertension management, adherence and pressure control in Blood Pressure Success Zone Program participants

**DOI:** 10.1111/j.1742-1241.2008.01840.x

**Published:** 2008-09

**Authors:** K A Payne, J J Caro, W L Daley, Z M Khan, K J Ishak, K Stark, D Purkayastha, J Flack, E Velázquez, S Nesbitt, D Morisky, R Califf, for the BPSZ-BLISS Study Steering Committee

**Affiliations:** 1 United BioSource Corporation, Health Care AnalyticsMontreal, QC, Canada; 2 United BioSource Corporation, Health Care AnalyticsConcord, MA, USA; 3 Division of General Internal Medicine, McGill UniversityMontreal, QC, Canada; 4 sanofi-aventis, Cardiovascular/ThrombosisBridgewater, NJ, USA (formerly with Novartis Pharmaceuticals, East Hanover, NJ, USA); 5 Novartis Pharmaceuticals, Department of Pricing Strategy and PolicyEast Hanover, NJ, USA; 6 Novartis Pharmaceuticals, Department of US Clinical Development/CardiovascularEast Hanover, NJ, USA; 7 Novartis Pharmaceuticals, Department of US Clinical Development/BiometricsEast Hanover, NJ, USA; 8 Department of Internal Medicine, Wayne State UniversityWayne, NC, USA; 9 Department of Medicine, Duke UniversityDurham, NC, USA; 10 University of TexasDallas, TX, USA; 11 University of CaliforniaLos Angeles, CA, USA; 12 Duke University, Translational Medicine InstituteDurham, NC, USA

## Abstract

**Aims:**

The Blood Pressure Success Zone (BPSZ) Program, a nationwide initiative, provides education in addition to a complimentary trial of one of three antihypertensive medications. The BPSZ Longitudinal Observational Study of Success (BPSZ-BLISS) aims to evaluate blood pressure (BP) control, adherence, persistence and patient satisfaction in a representative subset of BPSZ Program participants. The BPSZ-BLISS study design is described here.

**Methods:**

A total of 20,000 physicians were invited to participate in the study. Using a call centre supported Interactive Voice Response System (IVRS), physicians report BP and other data at enrolment and every usual care visit up to 12 ± 2 months; subjects self-report BPs, persistence, adherence and treatment satisfaction at 3, 6 and 12 months post-BPSZ Program enrolment. In addition to BPSZ Program enrolment medications, physicians prescribe antihypertensive medications and schedule visits as per usual care. The General Electric Healthcare database will be used as an external reference.

**Results:**

After 18 months, over 700 IRB approved physicians consented and enrolled 10,067 eligible subjects (48% male; mean age 56 years; 27% newly diagnosed); 97% of physicians and 78% of subjects successfully entered IVRS enrolment data. Automated IVRS validations have maintained data quality (< 5% error on key variables). Enrolment was closed 30 April 2007; study completion is scheduled for June 2008.

**Conclusions:**

The evaluation of large-scale health education programmes requires innovative methodologies and data management and quality control processes. The BPSZ-BLISS design can provide insights into the conceptualisation and planning of similar studies.

**What's known**

Educating patients with hypertension can help improve blood pressure control rates.

**What's new**

This paper contributes a detailed description of a methodology to assess, naturalistically, real-world health outcomes in a usual care population of more than 10,000 patients participating in a large-scale health education initiative related to the management of hypertension.

## Introduction

Despite an abundance of antihypertensive medications with proven efficacy, up to two-thirds of treated hypertensive patients do not achieve blood pressure (BP) control (1,2). The failure to take medications according to the prescribed regimen (non-adherence), and even worse, the discontinuation of treatment (non-persistence) are the main factors contributing to this problem (3–9). Observational data from community practice reveal that non-persistence with antihypertensive medications is a significant problem (10). Up to 25% of patients with hypertension discontinue their medications within months of their first prescription (11,12) – a modifiable behaviour (6,10) that has serious clinical (13,14) and economic (15) consequences. Failure to control hypertension in the USA has been estimated to cost US $467 million among treated patients and US $964 million if both treated and untreated are considered (16). In Canada (17) and the USA (18), the estimated average annual medical cost per patient for the treatment of hypertension is approximately $4000 USD. Given the cost and the medical implications associated with uncontrolled BP, the US government has embarked on a national initiative (2010 initiative) to control 50% of treated hypertensive patients by the year 2010 (19).

That so many patients risk serious and costly cardiovascular complications by discontinuing or misusing their medications (20), suggests that increased patient support and education could improve outcomes. Recent studies have demonstrated that educating patients about their health, the consequences of hypertension, and ways to optimise treatment can improve BP control rates (21–25). A recent review of 56 randomised clinical trials that determined the effectiveness of interventions to improve BP control noted that the methodological quality of these studies were poor to moderate, and indeed only one trial, the Hypertension Detection and Follow-up study was conducted on a large-scale real-world population (26).

The Blood Pressure Success Zone (BPSZ) Program is a comprehensive, patient-centred, treatment, education and support programme designed to improve persistence and adherence with antihypertensive medication. The BPSZ Program, offered to more than 40,000 US physicians since July 2005, will enrol more than 100,000 patients with newly diagnosed or uncontrolled chronic hypertension. The BP Success Zone Program was designed by behavioural psychologists based on a proven model of self-efficacy, or the belief that one's own health behaviours are under one's own control.

Patients can join the BP Success Zone Program once they have received an enrolment kit from their doctor, which includes a complimentary product of either benazepril/amlodipine fixed combination (Lotrel®; Novartis Pharmaceuticals Corporation, East Hanover, NJ, USA), valsartan (Diovan®; Novartis Pharmaceuticals Corporation) or valsartan/HCTZ (co-Diovan®), selected at the discretion of the physician for the trial of up to 30 days. The enrolled patients can avail up to $10 off of a first prescription for any of these products or a 14-day voucher, a $40 rebate for an Omron® BP monitor (Omron Corporation, Kyoto, Japan), a guarantee of reaching BP goal or getting the first 4 months of out-of-pocket prescription costs refunded, patient education delivered via mailings customised to participant demographics and attitudes and behaviours, as well as web tools to help manage their hypertension. When patients enrol in the BPSZ Program, they can choose among one of five Healthy Life-Style Action Plans, each of which consists of five communications to the patient via print or e-mail. The communications are in the form of newsletters that educate patients on that particular plan.

Although prior evidence (21–25) suggests that an initiative such as the BPSZ Program will contribute positively to patient health, to date, clinical outcome data have not been collected. These data are essential, however, for quantifying the impact of this programme. The main objective of the BPSZ Longitudinal Observational Study of Success (BPSZ-BLISS), therefore, was to evaluate BP control, adherence, persistence, and patient satisfaction as naturalistically as possible in a representative subset of the BPSZ Program participants. The objective of this manuscript is to describe the design and methods of the BPSZ-BLISS study.

## Methods

### Background

The BPSZ Program, which is still ongoing, has been offered to more than 100,000 patients with hypertension in the usual care setting to help achieve and maintain a BP goal as recommended by the JNC-VII guidelines (27). Physicians invite patients from their medical practices to participate in the BPSZ Program and provide interested patients with a Program Kit (Novartis Pharmaceuticals Corporation) to enrol. The Program Kit includes some customised educational materials, a guarantee of reaching the BP goal or getting the first 4 months of out-of-pocket prescription costs refunded, a $10 coupon for the first prescription, a $40 rebate for an Omron® BP Monitor and up to 30 days of medication [either benazepril/amlodipine fixed combination (Lotrel®), valsartan (Diovan®) or valsartan/HCTZ (co-Diovan®) selected at the discretion of the physician].

To enrol in the BPSZ Program, patients must be willing to receive a trial prescription for either benazepril/amlodipine fixed combination, valsartan, or valsartan HCTZ alone or in combination with other antihypertensive medications. Patients must also be new to the programme and have either a new diagnosis of hypertension (antihypertensive naïve patient), or an established diagnosis of hypertension that is uncontrolled on antihypertensive medication. The diagnosis of lack of control is defined as systolic BP ≥ 140 mmHg and/or diastolic BP ≥ 90 mmHg except in the presence of diabetes or renal disease where the limits are 130 mmHg for systolic and 80 mmHg for diastolic BP. By design, in the BPSZ-BLISS study, comparisons will not be made with regards to differences in efficacy or effectiveness of specific antihypertension medications. More importantly, to avoid the perception of bias by the sponsor, multiple checks and balances have been implemented including oversight by an academic Steering Committee.

Within 18 months of launch of the study, over 700 IRB approved physicians consented and enrolled 10,067 eligible subjects (48% male; mean age 56 years; 27% newly diagnosed); 97% of physicians and 78% of subjects successfully entered Interactive Voice Response System (IVRS) enrolment data. Automated IVRS validations maintained data quality (< 5% error on key variables). Enrolment was closed on 30 April 2007; the study was completed in June 2008. Details of the design and methods of the BPSZ-BLISS study are presented here. More detailed baseline characteristics of the study cohort will be reported in a separate publication.

### BPSZ-BLISS design overview

Blood Pressure Success Zone-BLISS was designed to evaluate the effect of the BPSZ Program on key outcome measures such as the proportion of patients achieving BP control, medication persistence, treatment adherence and satisfaction. This observational, multicentre study was approved by a central ethics review board. The only study-specific enrolment criteria, which differed from those of the BPSZ Program, were the need to be fluent in either English or Spanish and, that study subjects had a functioning touch-tone telephone.

To enrol in the BPSZ-BLISS study, subjects must have provided informed consent and then enrolled themselves in the BPSZ Program as per Program guidelines. Study physicians must also have activated the study identification number of each consenting subject in the BPSZ-BLISS database. Subjects may have voluntarily withdrawn from the study at any time but withdrawal did not preclude ongoing participation in the BPSZ Program. A minimum of 10,067 subjects, approximately a 10% sample of BPSZ Program participants, were enrolled to allow for the estimation of BP control rates at various time points in sub-populations defined by age, gender, ethnicity, hypertension status (new vs. chronic) and presence of diabetes or renal disease.

Investigational drugs were not administered to study subjects, although at baseline, BPSZ Program medications must have been prescribed alone, or in combination with other concomitant antihypertensives, as per BPSZ Program enrolment criteria. Consistent with the observational design, however, treatment for hypertension was managed by the physician as per routine care following the baseline (enrolment) visit.

More specifically, choice of antihypertensive drug, dose, and treatment regimen in individual patients was determined by the managing physician throughout the study period. Following the baseline visit, BPSZ Program medications could have been removed from the treatment regimen at the discretion of the physician. Physicians were instructed to report serious adverse events as per standard medical practice.

To provide a reference for the BP control rates documented in BPSZ-BLISS, an external reference group (28,29) will be created by identifying a cohort of similar patients whose management is recorded in the General Electric (GE) Healthcare Electronic Medical Record (EMR) database. This is a comprehensive national dataset containing both clinical (e.g. BP) and administrative (e.g. MD visits) information (30).

### Recruitment and training

Approximately 20,000 physicians enrolled in the BPSZ Program were randomly selected from a list of 40,000 physicians who had previously participated in Novartis-sponsored hypertension health education initiatives and invited to participate in BPSZ-BLISS. The first 2000 physicians who expressed interest in participating completed the appropriate documents and underwent the study training to enrol patients. Contracting, IRB applications and IRB renewals were supported by an internet-based document management system (Synovate ViewsCast, Chicago, IL). Upon receipt of the IRB applications via the internet, physician information was provided to a central IRB agency (IRB Services, Aurora, Canada) responsible for study approvals.

Once IRB approved and trained, physicians were provided with BPSZ-BLISS materials. Physicians invited into the study only those patients who had already decided to participate in the BPSZ Program. Participating physicians were remunerated quarterly for a total of USD $200 per enrolled patient. So as not to encourage additional medical visits outside of usual care, payments were made quarterly in full irrespective of whether follow-up data were entered, or subjects had completed the study.

### Study conduct

#### Data collection and management

Blood Pressure Success Zone-BLISS data were collected and managed electronically using an IVRS – an integration of computer-automated interviewing with touch-tone telephone service (Synovate ViewsCast). The IVRS, available 24 h a day, 7 days a week, allows for entry of information directly into the electronic database. Call centre representatives were also available via the IVRS routing menu to assist callers in between data entry periods. Access to the IVRS was via a toll-free telephone number. To ensure security, a link was created by the physician during the baseline study visit using the participant's BPSZ Program identification number and date of birth.

To facilitate delivery of data entry reminders by the IVRS and call centre provider, patients must have consented to the inclusion of their name, home address and phone number. Only representatives of the IVRS call centre – not the study sponsor or its representatives – had access to this information. Patients who did not enrol in the BPSZ Program within 7 days of the BPSZ-BLISS enrolment visit were issued an automated telephone message. A similar reminder was issued to all subjects 3 days prior to each 3, 6 and 12 months visit. If data were not entered within 6 days of this prompt, then call centre representatives made a maximum of four attempts to reach the subject by phone over a 1-week period. With respect to the physician-reported data, the number and timing of healthcare consultations with subjects over the study period were not mandated by the protocol but physicians were sent generic reminders that study data should be entered into the IVRS following all routine care visits.

Physicians received prepackaged patient study boxes (one per subject) containing both the physician's worksheets, to be used during all visits to record study data, and all the materials a subject needed to participate. For example, each study subject received a box that included an instruction booklet (in English or Spanish), a complimentary BP monitor, a wallet card to record BP values, and a standard BPSZ Program kit, containing a 30 day sample of valsartan, valsartan HCTZ or benazepril/amlodipine fixed combination (selected and prescribed at the discretion of the physician), as well as a $10 coupon off for the first prescription. The kit containing medication was not tailored in any way for study participants. It was exactly the same kit that all BPSZ Program participants received even if they were not enrolled in the BPSZ-BLISS study.

#### BPSZ-BLISS study baseline (enrolment) visit

At the BPSZ-BLISS baseline enrolment visit, the physician removed a two-ply informed consent form and a set of worksheets (which parallelled the IVRS data entry script) from the study box and clipped them to the inside of the patient's medical chart. These worksheets were used during the baseline visit and at every usual care visit thereafter to record study data prior to contacting the IVRS.

After obtaining an informed consent from the patient, the physician initiated the BPSZ-BLISS study enrolment process by activating the patient's BPSZ Program membership identification number found on the BPSZ Program kit and, for security purposes, linked this number to the subject's date of birth. The following data were entered into the IVRS: most recent hypertension medication, dose and daily regimen (from last office visit, if applicable), current BP (from baseline study visit) and study enrolment antihypertensive medication(s) (valsartan, valsartan HCTZ or benazepril/amlodipine fixed combination, and other concomitant antihypertensives), dose(s) and daily regimen. Systolic and diastolic BPs were measured by study physicians using routine practice – no standardised method was mandated by the study protocol, although the type of BP monitor typically used at each physician's office was documented. During this visit, the subjects also measured their own BP using the complimentary home BP monitor and recorded these values in the wallet card provided.

#### BPSZ program patient self-enrolment

**Figure 1:**
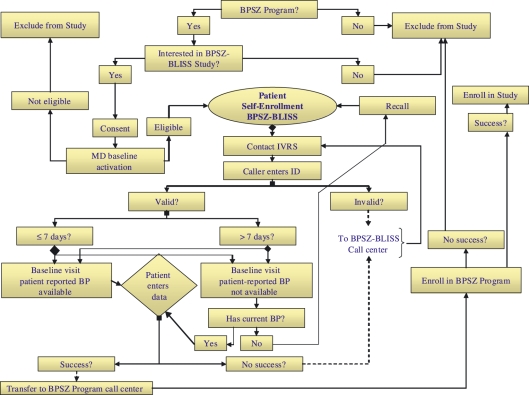
Overview of BPSZ-BLISS Study and BPSZ Program self-enrolment. BPSZ-BLISS, Blood Pressure Success Zone Longitudinal Observational Study of Success

To be eligible for the BPSZ-BLISS study enrolment process, patients must have self-enrolled into the BPSZ Program. To this end, subjects contacted the study IVRS, entered their subject identification number and date of birth, and then enter their patient-reported data before being transferred to the BPSZ Program enrolment call centre. Prior to this transfer, the patient was asked if he/she had started taking his/her new antihypertensive medications prescribed during the baseline visit, and if so, the date that the medication was started. Each subject also entered his/her systolic and diastolic BP measured by the subject during the enrolment visit. To ensure a relevant subject-reported enrolment BP, if the subject called to enrol in the programme more than 7 days poststudy enrolment, the subject was also asked to enter a BP reading obtained within the previous 7-day period. Upon completion of this IVRS segment, the subject was transferred to a live BPSZ Program agent (Figure 1). Once enrolled in the Program, in addition to BPSZ Program materials, subjects eligible for the study were also mailed a personalised BPSZ-BLISS study welcome letter and a magnet which specified the patient's actual 3-, 6- and 12-month calendar dates that study data should have been entered into the IVRS.

#### Follow-up visits

**Figure 2:**
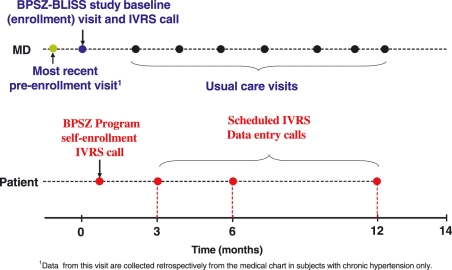
BPSZ-BLISS design overview. IVRS, Interactive Voice Response System; BPSZ-BLISS, Blood Pressure Success Zone Longitudinal Observational Study of Success

During every subsequent usual care visit over the course of the 12 (±2)-month study period, physicians also reported the patient's BP and any changes to the baseline antihypertensive medication(s) including dose, daily regimen and reason(s) for change such as contraindications, or the discretion of the physician. Patient height, weight and waist circumference were also measured and reported. All data were entered first on the appropriate worksheet and then entered into the IVRS. At 3, 6 and 12 months (−3, +10 days) postenrolment in the BPSZ Program, patients entered data related to BP, persistence with, and adherence to antihypertensive medication as well as satisfaction with treatment. If the patient indicated during the 3-, 6- and 12-month IVRS assessment that he/she had not stopped taking his/her antihypertensive medication, the patient completed the Modified Morisky Scale (seven items) (31,32), a measure of treatment adherence, using his/her own pattern of antihypertensive medication use as a reference. Patients still persistent at these time points also completed a modified version of the Treatment Satisfaction Questionnaire for Medication (TSQM) (33). In the modified questionnaire, only the TSQM effectiveness (items 1–3), convenience (items 9–11) and global satisfaction (items 12–14) sub-scales were administered. The TSQM is a psychometrically robust instrument, which taps into the most important dimensions of patients’ experiences with their medication. A manuscript reporting the psychometric properties of the modified scale is currently in preparation. The BPSZ-BLISS study assessment schedule is presented in Figure 2.

### Statistical analyses

Analyses will be performed on data obtained from patients who were successfully enrolled in the BPSZ-BLISS study and the BPSZ Program, and for whom data on BP measurements, as well as persistence, adherence and satisfaction to antihypertensive medication during follow-up was reported.

The study population will be characterised with respect to cohort disposition, the distribution of the age, gender, ethnicity, weight, height, waist circumference, prior comorbidities, baseline BP, hypertension status, diabetes status and treatment prescribed by the physician at the baseline study visit. These will be presented and summarised with appropriate descriptive statistics (mean, standard deviation, median and range for continuous variables; number and percentage for categorical variables).

Analyses will examine outcomes in the overall population as well as within subgroups defined by gender, age (e.g. 18–44 years of age; 45–54 years of age; 55–64 years of age and ≥ 65 years of age), ethnicity (e.g. Caucasian; African American; Hispanic and other), hypertension status (newly diagnosed vs. chronic patients) and other prior comorbidities.

Primary analyses of BP control will be based on physician-reported data. Definition of BP control will be based on the JNC-VII guidelines (Table 1) (27). Imputation, or other appropriate methods, will be undertaken if a high rate of missing BP measurements is found. Last-observation-carried-forward, linear interpolation and multiple imputation will be considered, depending on the patterns of missing data. The proportion of patients who achieved BP control over time will be described and summarised using Kaplan–Meier curves for the overall population and subgroups. Differences between subgroups will be tested using the log-rank and Wilcoxon tests. A proportional hazards model will be fitted to identify predictors of time to control. Alternative measures of BP control, such as time to control, or change from baseline will also be examined.

**Table 1:** Summary of blood pressure control definitions (27)

BP classification	SBP (mmHg)		DBP (mmHg)
Normal	< 120	and	< 80
Prehypertensive	120–139	and/or	80–89
Stage 1 hypertension	140–159	and/or	90–99
Stage 2 hypertension	≥ 160	and/or	≥ 100

BP, blood pressure; SBP, systolic blood pressure; DBP, diastolic blood

Data on persistence, adherence and satisfaction were reported by patients at 3, 6, and 12 months. At each time point, the number and proportion of patients will be described for patients still followed in the study and by subgroups. Adherence and satisfaction with treatment will be described for patients who are persistent at months 3, 6 and 12 months. Adherence is measured with the Morisky Scale (31,32), with scores ranging from zero to seven. A modified version of the TQSM (33) is used to evaluate satisfaction with total effectiveness, convenience and global satisfaction, with scores ranging from 0 to 100 for each sub-scale. The distribution of scores will be described in terms of the mean, standard deviation and quartiles in the overall population and by subgroups. The proportion reporting high, moderate or low adherence (defined as Morisky scale score of 7, 5 to <7, <5 or below, respectively) and satisfaction will also be described. Given the volume and scope of data related to patient demographics and the management of hypertension, which will be collected in BPSZ-BLISS, numerous analyses related to usual care management patterns by physician and/or patient characteristics are also anticipated.

## Conclusions

The BPSZ-BLISS study was designed to evaluate the impact of the BPSZ Program, a prospective intervention for the management of hypertension using an external reference control group. This design aims to achieve a balance between the need for some control to properly collect data from a large real world population of patients and the need to collect these data, naturalistically, by minimising interference with physician and subject behaviour. As such, observational and naturalistic principles are central to the BPSZ-BLISS study design. Apart from prescribing the BPSZ Program antihypertensive medication alone or in combination with other concomitant antihypertensive medication at the baseline visit, physicians were free to administer additional or replacement antihypertensive medications, adjust dosing regimens and schedule office visits at their professional discretion. Study physicians were instructed to evaluate BP and other clinical variables as per routine care – no standardised methods were imposed. On the patient side, to minimise response bias, patients responded to questions related to BP, persistence, adherence and treatment satisfaction via the IVRS, far and away from the potential influence of their healthcare professionals who treat them.

One of the important features of the BPSZ-BLISS study design is that it permitted the collection of BP values from both physicians and patients over a 10- to 14-month period. Data such as these will allow for analyses of BP control rates in actual practice resulting in estimates of proportions of patients controlled and time to control (6). The comparison of physician- and patient-measured BPs, given that both were assessed and collected in this study, will contribute to a better understanding of ambulatory BP management. A concern that might be raised is that patients enrolled in the BPSZ Program are prescribed an initial regimen that includes an antihypertensive drug made by the sponsor. To guard against a potential bias in the interpretation of study results, various checks and balances were employed. For example, the analyses of study data will not involve any drug comparisons with regard to efficacy or effectiveness. The rates of use of different drugs and combinations will be reported as a component of the ecology of the intervention, but comparisons with regard to BP lowering or clinical events will not be made. These medications are just one feature of the comprehensive BPSZ Program, and it will not be possible to evaluate with any validity the relative impact of the various programme components (e.g. enrolment visit medication vs. educational materials etc.) on the outcomes of interest. Furthermore, an academic steering committee was assembled to provide oversight for the study, the analytical methods employed, the interpretation of results, as well as all publications. Funding sources and potential conflicts of interest have been made transparent. Clearly, these efforts do not completely diminish the risk of bias, but in our view, the evaluation of effectiveness that the BPSZ-BLISS study provides is no different in this regard than an RCT in which the sponsor's drug is a comparator.

To put the outcomes of the BPSZ-BLISS results in context, the GE EMR database (30) has been selected as a suitable source of external reference data. Limitations to externally controlled studies exist, however. For example, it is difficult to ensure that both the treatment and control groups are comparable in terms of demographic and clinical features, diagnostic criteria, disease severity and concomitant treatments – hence bias may be a problem. Moreover, time as a determinant of outcomes must also be considered given the potential for changes in treatment patterns, guidelines and available treatments. Statistical issues may be complex as techniques such as regression analyses, propensity scores or selective sampling must be employed to deal with missing data and to control confounding variables as much as possible. The persuasiveness of findings depends on a much larger difference in treatment effect and higher levels of statistical significance than would be considered necessary in a concurrently controlled trial. Limitations aside, in many circumstances such as this study, a perfect control group may be impossible to achieve. A separate and subsequent publication will describe in detail the rationale underlying this approach and the methods, which will be employed.

A main premise of this design is that BPSZ-BLISS study results derived from a 10% sample of BPSZ-Program participants can be generalised to the BPSZ Program population as a whole. Accordingly, demographic and clinical characteristics must be shown to be comparable, and the potential impact of observer effects on hypertension management outcomes in the study cohort must be explored. With respect to population characteristics, study selection criteria were designed to match that of the BPSZ Program and additional restrictive criteria common to randomised and controlled trials were not employed. Further, study participants were enrolled from all states that offer the BPSZ Program and physicians were trained to recruit for the study only those patients who indicated first that they wanted to enrol in the BPSZ Program. Regarding observer effects, although BPSZ-BLISS subjects provided informed consent, a reminder to them that they were being studied, consent must also have been provided by all patients enrolling in the BPSZ Program, even if they were not participating in the study. In general, while the Hawthorne effect (i.e. the impact on behaviour of the awareness of being observed) may alter study patient and physician behaviour to some degree, significant observer effects are also present in the BPSZ Program itself. In fact, the global aim of the BPSZ Program is to make patients acutely aware of the medical services and treatments that are available to them and to remind them on an ongoing basis of the need for various lifestyle interventions. Programme participants receive a series of tailored mailings, which promote healthy behaviours and encourage patients to communicate regularly with their healthcare provider. Thus, while it is impossible to completely eliminate the influence of observation on study outcomes or even measure this phenomenon, in theory, the differential impact of observer effects on study participants vs. the same in non-study BPSZ Program participants may actually be minimal given the nature of the BPSZ Program intervention.

Data collected as a result of the BPSZ-BLISS study have resulted in a rich dataset that will support diverse analyses of usual care hypertension management and related clinical and patient-reported outcomes. Given the very large sample size and inherent ethnic diversity of the study cohort, BPSZ-BLISS provides an opportunity for numerous subgroup analyses of interest, defined both in terms of physician and subject characteristics. BPSZ-BLISS data will also support the testing and further development of analytical methods applicable to longitudinal datasets that reflect a routine care assessment schedule. Finally, although the BPSZ-BLISS design has been tailored to collect data on the outcomes from patients with hypertension, the main parameters, infrastructure and methodologies employed could be adapted to the evaluation of any large-scale health improvement intervention. At this time, 10,067 eligible patients have consented and been enrolled in the study by over 700 participating physicians. Although study enrolment closed in April 2007, and data collection terminated in June 2008.
